# A Self-Powered and Battery-Free Vibrational Energy to Time Converter for Wireless Vibration Monitoring

**DOI:** 10.3390/s21227503

**Published:** 2021-11-11

**Authors:** Namanu Panayanthatta, Giacomo Clementi, Merieme Ouhabaz, Mario Costanza, Samuel Margueron, Ausrine Bartasyte, Skandar Basrour, Edwige Bano, Laurent Montes, Catherine Dehollain, Roberto La Rosa

**Affiliations:** 1 Institut IMEP-LaHC, University Grenoble Alpes, University Savoie Mont Blanc, CNRS, Grenoble INP, IMEP-LaHC, 38000 Grenoble, France; namanu.panayanthatta@grenoble-inp.org (N.P.); edwige.bano@grenoble-inp.fr (E.B.); Laurent.Montes@grenoble-inp.fr (L.M.); 2 FEMTO-ST Institute, University of Bourgogne Franche-Comté, CNRS (UMR 6174), ENSMM, 26 rue de l’Epitaphe, 25030 Besançon, France; giacomo.clementi@femto-st.fr (G.C.); merieme.ouhabaz@femto-st.fr (M.O.); mario.costanza@femto-st.fr (M.C.); samuel.margueron@femto-st.fr (S.M.); ausrine.bartasyte@femto-st.fr (A.B.); 3 TIMA, University Grenoble Alpes, CNRS, Grenoble INP, 38000 Grenoble, France; skandar.basrour@univ-grenoble-alpes.fr; 4 Ecole Polytechnique Federale de Lausanne, 1015 Losanne, Switzerland; catherine.dehollain@epfl.ch; 5 STMicroelectronics, Stradale Primosole 50, 95121 Catania, Italy

**Keywords:** energy harvesting, piezoelectric transducers, vibrational sensors, internet of things (IoT), low power, microcontroller, Bluetooth low energy, wireless sensor network, wireless sensor node

## Abstract

Wireless sensor nodes (WSNs) are the fundamental part of an Internet of Things (IoT) system for detecting and transmitting data to a master node for processing. Several research studies reveal that one of the disadvantages of conventional, battery-powered WSNs, however, is that they typically require periodic maintenance. This paper aims to contribute to existing research studies on this issue by exploring a new energy-autonomous and battery-free WSN concept for monitor vibrations. The node is self-powered from the conversion of ambient mechanical vibration energy into electrical energy through a piezoelectric transducer implemented with lead-free lithium niobate piezoelectric material to also explore solutions that go towards a greener and more sustainable IoT. Instead of implementing any particular sensors, the vibration measurement system exploits the proportionality between the mechanical power generated by a piezoelectric transducer and the time taken to store it as electrical energy in a capacitor. This helps reduce the component count with respect to conventional WSNs, as well as energy consumption and production costs, while optimizing the overall node size and weight. The readout is therefore a function of the time it takes for the energy storage capacitor to charge between two constant voltage levels. The result of this work is a system that includes a specially designed lead-free piezoelectric vibrational transducer and a battery-less sensor platform with Bluetooth low energy (BLE) connectivity. The system can harvest energy in the acceleration range [0.5 g–1.2 g] and measure vibrations with a limit of detection (LoD) of 0.6 g.

## 1. Introduction

The recent advent of Internet of Things (IoT) technology has had a direct and marked impact on the industrial, healthcare, agricultural, automotive, smart homes, and many other sectors [1,2,3]. IoT infrastructure makes wide use of WSNs that can simultaneously function as sensors, communication devices, and information processors [4,5]. WSNs rely primarily on bulky and expensive batteries with limited charge/discharge cycles that to some extent hinder a more widespread implementation due to costly and labor-intensive maintenance requirements and, since battery storage capacity depends on volume, they can also introduce size and weight issues. Furthermore, in heavy machinery, these nodes are often placed in difficult-to-reach positions where battery replacement is problematic and contributes to increasing asset management costs and complexity. Energy-autonomous and battery-free WSNs therefore represent highly favourable solutions for sustainable and autonomous IoT systems, where possible. In this context, energy-autonomous wireless sensors (EAWSs) are a promising solution for boosting the deployment of IoT systems, as they facilitate the deployment of efficient, low-maintenance, ubiquitous, set-and-forget monitoring systems and contribute to a substantial reduction in the number of battery disposals [6]. Significant interest and research has been invested by the scientific community into battery-free solutions that exploit the energy collected from the environment through different energy sources such as solar, vibration, thermal, and radio-frequency waves, etc., in combination with small capacitors that are intermittently charged and discharged to power the WSN [7,8,9,10,11]. In automotive and avionics industries that use heavy machinery, the failure of components such as rotating shafts can cause severe damage during operation [12,13]. Wireless and energetically autonomous sensors can address these issues in predictive maintenance scenarios based on characteristic trend analyses of vibration data collected by the sensors around mechanical parts in motion. A non-contact vibration monitoring technique employing WSNs could prove especially advantageous in this scenario over more conventional methods involving contact between the sensor and control system. Non-contact techniques to date have been based on magnetostrictive sensors [13,14] and optical sensors [15]. Such non-contact sensors, however, are limited in their dependence on external battery sources and sensitivity to harsh operating environments [16]. The use of batteries can in some cases be avoided by introducing an energy harvester that exploits mechanical, solar, thermal, electromagnetic, RF, vibration or similar energy sources around the WSN [17,18,19,20]. Recent studies have proposed a triboelectric nanogenerator as the main energy source for self-powered acceleration sensors [21,22]. Further, piezoelectric transducers are also one of the most convenient ways to convert ambient mechanical energy into electrical energy, as piezoelectric harvesters (PEH) are relatively simple to manufacture and have better power densities than most other conversion techniques, including RF, thermal or electrostatic methods [23]. Piezoelectric energy harvesters (PEHs) can be simple, single-clamped cantilever beams with a piezoelectric material and a substrate. This type of device is employed in this study to function as a self-powered sensor simultaneously able to monitor vibration phenomena and power data transmission circuitry [24,25,26]. This paper introduces an energy-autonomous wireless vibration sensor (EAWVS) for monitoring vibrations. Since defective components in machinery have a more pronounced acceleration profile than non-defective components [27], this work develops a failure detection methodology based on the deviation of acceleration measurements from nominal values associated with non-defective componentry. The system can detect vibrations from a minimum limit of detection (LoD) acceleration of 0.6 g, effectively representing an EAWVS consisting of only a PEH for power supply and a Bluetooth low energy (BLE) radio for connectivity. The sensor node can be connected via wireless RF to a base station (BS) that performs data processing and streaming to the cloud. The BLE communication standard was chosen due to its low power consumption and relatively seamless integration of portable devices in BLE WSNs. However, the sensing method proposed in this paper does not depend on the adopted communication system. Nothing prevents adopting another communication system such as LoRa, SigFox, Wifi, Zigbee, and the like provided that the power consumption is compatible with the use case, the sensor duty cycle, and available power generated by the harvester. The sensor node is designed to transmit beacons intermittently, with the radio shutting down after each transmission to minimize energy consumption. The special feature of this system is that the PEH performs the dual-role of an energy transducer and vibration sensor. Input vibration is monitored by measuring the time between successive beacons, also known as the advertising time ($t_{adv}$), emitted by the EAWVS radio to the BS. This method eliminates the need for a specific sensor and carries major benefits in terms of power consumption and consequent energy storage requirements. The system consists of fewer components and the absence of any battery or super-capacitors ensures significant cost, dimension and weight reductions. State-of-the-art high-performing PEHs are manufactured on lead-based materials such as PbZr${}_{1-x}$Ti${}_{x}$O${}_{3}$ (PZT) [28,29], which although capable of achieving considerable power densities (1039 μW/cm${}^{2}/$g${}^{2}$ [30]), contain the toxic Pb element that conflicts with REACH (Registration, Evaluation, Authorisation and Restriction of Chemicals) and RoHS (Restriction of Hazardous Substances) regulations. For this reason, the electronics industry is shifting to environmentally friendly materials and processes for next-generation “green” PEHs [31]. Indeed, innovative lead-free materials and structures represent tremendous challenges and opportunities for energy harvesters and self-powered sensors, and continue to gain interest in scientific circles [32,33,34,35]. Concerning piezoelectric LiNbO${}_{3}$ (lithium niobate—LN), it was demonstrated in [36] that the electro-mechanical properties of single LN crystals are highly dependent on orientation, and are comparable with PZT ceramics. An initial demonstration was performed at high frequency and acceleration (1.14 kHz and 3.4 g), with the material integrated on silicon cantilevers in an optimized design approach including an electronic interface, and the harvester was able to attain 71 μW/cm${}^{2}/$g${}^{2}$ at the resonance frequency and form a wireless connection with an RF sensor [37]. Recently, flexible cantilevers with a tip mass based on 128${}^{\circ }$Y-cut LN on Si have been able to achieve a power density of 965 μW/cm${}^{2}/$g${}^{2}$ at 0.1 g and 105.9 Hz resonance frequency [38]. This result is among the highest with respect to both Pb-based and Pb-free current devices. In this context, this paper also aims to contribute to the study of the widely known lead-free material, LN, in vibrational energy-harvesting applications with a design proposal to increase cantilever sensitivity at lower acceleration and frequencies with a large tungsten tip mass. The aim is to match the typical frequencies and acceleration characteristics of heavy machinery and, together with an optimized BLE connectivity system, deliver a viable EAWVS solution with a resonance frequency of 187 Hz.

The paper is organized as follows: Section 3 describes the system in terms of circuit implementation and functionality; Section 4 deals with the design aspects of the piezoelectric energy harvester; Section 5 covers the techniques and the method based on energy to time conversion used to measure a vibration’s intensity from the magnitude of vibrational energy harvested by the EAWSN; Section 6 describes the system setup and environment and presents some experimental results; Section 7 reports the final conclusions.

## 2. Related Work

The IoT wireless node has evolved from a simplistic tag transponder used mainly for item identification to an advanced device that is also able to perform sensing and computations simultaneously. Indeed, the evolved node needs more energy, and typically uses a battery that eventually needs to be replaced periodically, incurring increased maintenance costs for wireless sensor networks. In this context, the ubiquitous nature of energy-autonomous WSNs, which are easy to install and simple to connect to a wireless sensor network, makes them attractive in the rapidly growing IoT ecosystem [39]. All this has aroused much interest in the scientific community by stimulating the production of research and scientific publications. Still, to date, issues related to the power transducer and energy storage devices, power management integrated circuits and radio communication protocols stop the evolution of energy-autonomous wireless sensor nodes from the phase of study and experimentation to use in practical applications available on the market. These issues are highlighted in [40], which presents a comprehensive review of the progress made in the study of piezoelectric (PENG) and triboelectric (TENG) nanogenerators. This paper shows how PENGs and TENGs can be used both as active sensing devices and power sources. In practical applications, the energy availability is highly dependent on the use case, which implies that different strategies and energy sources and systems must be used on a case-by-case basis. To this purpose, the authors of [6] show how to use diverse methods for powering wireless sensor nodes through energy harvesting and wireless power transfer. In general, piezoelectric materials have received considerable attention [41,42] in various fields due to their ability to convert mechanical energy into electrical energy. Indeed, there are also examples of interesting practical use cases such as the self-powered wireless sensor for smart and autonomous pavement described in [43]. Further, ref. [44] describes a self-powered sensor that integrates an energy harvester and a wireless sensing system. The energy harvester converts pressure fluctuations in hydraulic systems into electrical energy using an acoustic resonator, a piezoelectric stack, and an interface circuit. A high-performance and ultra-low-power management IC is a fundamental component in self-powered systems, and the recent advances in ultra-low power chip design techniques have enabled deep research into long-lifetime WSNs. On this topic, ref. [45] presents a fully integrated dc-dc converter topology based on electromagnetically coupled class-D LC oscillators that enable up to 2.5 GHz switching frequency, allowing aggressive scaling of the on-chip passives. Ref. [6] proposes an ultra-low-power 2.5 μW highly integrated mixed-signal system on chip for multi-source energy harvesting and wireless power transfer. Ref. [46] presents a multi-source 6.45 μW self-powered SoC for solar and thermoelectric generator (TEG) energy harvesting and [47] shows a developed chip to scavenge energy from artificial light. Energy storage devices are a fundamental part of systems supplied through energy harvesting. To date, these devices still require improvement as their quality and performance affect the overall system performance. A contribution to this research is in [48], which shows a study of the limitations of the energy storage devices, i.e., super-capacitor and lithium-polymer batteries, commonly used in energy-harvesting applications. A WSN must communicate wirelessly with other nodes within a wireless sensor network. The communication unit of a WSN is the most power-hungry part of the system, especially in wide-area networks where long-range communication is required. The authors of [8] contribute to this topic by explaining the challenges posed by combining low-power and long-range connectivity and evidence the dominance of the transmission over the quiescent energy while outlining the principles for achieving both low-power and wide-area communication. In some use cases, through careful design at the system level, the energy source can be used simultaneously to perform sensing or measurements with the advantage of having fewer components to power, e.g., the sensors, therefore ensuring a lower power consumption of the system and less stringent specifications relating to energy storage. One example of such a system is shown in [10], which describes a WSN for identifying and monitoring the speed of assets in a wireless sensor network with battery-less nodes combined with and a unique measurement approach to generate time-domain speed readouts. Ref. [7] proposes a wireless sensor platform with a single photovoltaic transducer that performs the dual role of harvesting energy and sensing ambient light. The authors show how the dual role played by the harvester/sensor allows smaller and cheaper nodes with a reduced component count. The current applications supplied through energy harvesting or wireless power transfer aim to replace toxic and non-eco-friendly energy sources, e.g., batteries, with more sustainable ones. The future of energy harvesting will be nature-driven, and the trend is to use non-toxic, biodegradable materials. Research on lithium niobate PEHs represents a further contribution towards green computing in wireless sensor networks [49] and ultimately a green internet of things [37,38]. Furthermore, refs. [50,51] systematically review effective ways of addressing green applications and energy-neutral systems, which operate with the energy harvested from their environment.

## 3. System Description

**Figure 1:**
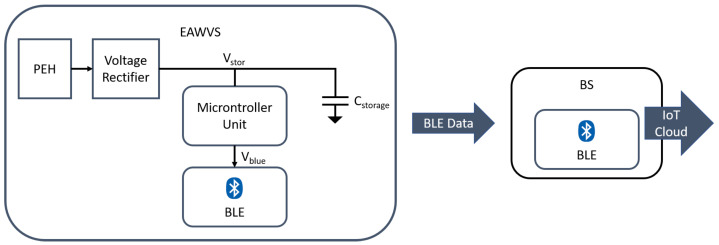
System block diagram EAWVS/BS.

Figure 1 depicts the block diagram of the system consisting of the following units:

- The energy-autonomous wireless vibration sensor (EAWVS): the main requirement of this block is to be energy-autonomous and battery-free.
- The base station (BS): This device is powered by a stable power source such as a battery or wired power supply. Its function is to process the incoming wireless data from one or more EAWVSs and process the subsequent implementation phases to control the machinery, including sending the data to the cloud.

The EAWVS consists of a specially designed PEH to transduce input mechanical vibrations into electrical energy stored in the storage capacitor $C_{storage}$. The PEH supplies the energy stored in the storage capacitor and also acts as the transducer for the sensing activity. In this implementation, the harvested AC voltage is rectified using a full-bridge voltage rectifier and is used to charge the capacitor $C_{storage}$. The energy stored in the capacitor powers the BLE radio to transmit data from the EAWVS in advertising non-connectable mode. The system implements a programmable voltage detector (PVD) circuit that detects the voltage $V_{stor}$ across the storage capacitor $C_{storage}$ with the help of an STM32L0 ultra-low-power microcontroller (STMicroelectronics) [52]. It is worth noting that the use of the STM32L0 microcontroller is to uniquely exploit its ultra-low-power analog IPs, such as the power voltage detector (PVD) and the ultra-low-power timer (LPTIM). Indeed, the ultra-low-power mode functionalities of the STM32L0 microcontroller, such as the stop mode, are fundamental in this kind of application to achieve the lowest quiescent power consumption during the harvesting phase, ≈2.5 μW. The quiescent power is a fundamental parameter as it is the minimum power necessary to operate the system and ultimately defines its LoD. Reducing the quiescent power consumption as much as possible is essential in energy-harvesting applications to obtain lower values of LoD and, therefore, to be able to carry out a smaller implementation or operate with tenuous vibrations.

The EAWVS also includes the HTS221 (STMicroelectronics) temperature and humidity sensors on the board. These sensors are powered and interrogated every time the BLE sends a beacon. The data packet sent during a beacon also contains information on humidity and temperature. There is nothing to prevent the inclusion of other sensors on the EAWVS if the power consumption is compatible with the power generated by the vibrational transducer and the size of the storage capacitor that also defines the size and system form factor. In this specific case, given the low consumption of the humidity and temperature sensor IC, it was possible to perform sensing and data transmission in a single action. When the power consumption of the sensors is so high as to not allow it to carry out the two phases simultaneously, the solution adopted is to carry out solely the sensing phase, store the data in the memory of the microcontroller and then send them in the next beacon.

The BS uses the same BLE radio device as the EAWVS, but configured in scanning mode. Both the EAWVS and the BS effect BLE communication through the low-energy system on chip (SoC) BLUENRG-2 (STMicroelectronics) [53]. The widespread availability of BLE connectivity in mobile devices also greatly facilitates humans being able to interface with the node.

**Figure 2:**
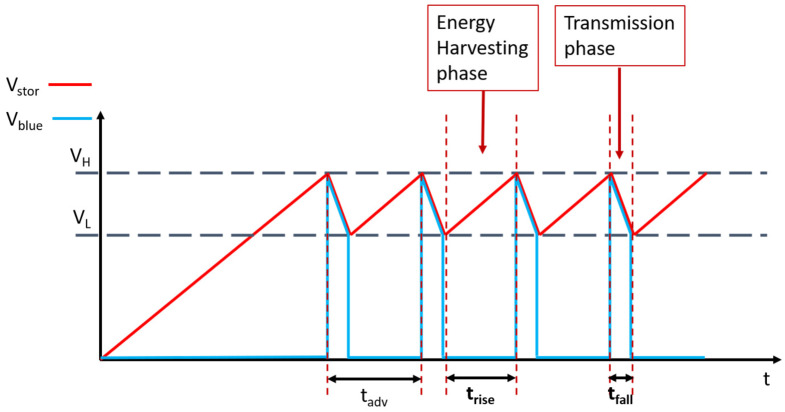
Functional evolution of the voltage $V_{stor}$ and $V_{blue}$ vs. time.

Figure 2 shows that as soon as the voltage $V_{stor}$ reaches the highest value $V_{H}$ = 3.0 V, the system enters the transmission phase, where the microcontroller supplies the voltage $V_{blue}$ to the BLE through one of its general-purpose input/output (GPIO) pins. Shortly after the transmission phase, when the voltage drops to the lowest value $V_{L}$ = 2.0 V, the microcontroller shuts down the BLE radio and enters the stop mode, during which the whole system resumes its energy-harvesting activity. The sensor method exploits the proportional relationship between the elapsed time between two successive beacons and the acceleration imparted on the PEH. The values of $V_{L}$ and $V_{H}$ are chosen such that the voltage never breaches the rated maximum (3.6 V) or minimum (1.6 V) supply voltage of the microcontroller. The power requirements of the microcontroller and BLE SoC, the settings of the BLE radio in terms of operation mode, transmitted power, and length of the data packet have already been described in detail in [7,54]. The paper [7] shows a detailed description of how the communication protocol between the EAWVS and the BS minimizes data collisions and flooding of the network in which they operate. Since the minimum energy requirement $E_{harvested}$ of the EAWVS is ≈ 100 μJ per beacon, the minimum value of the storage capacitor $C_{storage}$ can be determined by (1):

(1) $$C_{storage\_min}=\frac{2\;\cdot \;E_{harvested}}{V_{H}^{2}-V_{L}^{2}}=40\;\mu F.$$

To allow some margin and extra energy capability to optionally activate the other embedded sensors (HTS221) and thereby render system performance less sensitive to inevitable parametric variations of the various components involved, the chosen value for the $C_{storage}$ capacitor was 200 μF. This choice, together with the selected values of the voltages $V_{H}$ and $V_{L}$, allows the harvesting of a quantity of energy per cycle as given by Equation (2):

(2) $$E_{harvested}=\frac{1}{2}\;\cdot \;C_{storage}\;\cdot \;\left(V_{H}^{2}-V_{L}^{2}\right)=500\;\mu J/\mathrm{cycle}.$$

## 4. Piezoelectric Energy Harvester Design and Fabrication

The PEH is designed to meet the typical vibration levels of most transportation and industrial environments (cars, trains, heavy machinery, etc.), with typical frequency peaks between 100 Hz and 300 Hz, and with an acceleration of 0.1 g up to 2 g [55]. The device is therefore designed to meet the application specifications in terms of resonance frequency ($f_{0}$ = 187 Hz) and acceleration levels (0.5 g–1.2 g), with the aid of LiNbO${}_{3}$ high-quality piezoelectric single crystals, in order to optimize the electro-mechanical conversion. The PEH must produce a minimum power $P_{PEH\_min}$ to supply the EAWVS during the energy-harvesting phase under the minimum vibrational acceleration $a_{cc\_min}$, which can represent the limit of detection (LoD) of the system in its function as a vibration sensor. To ensure that the capacitor $C_{storage}$ is effectively charged from $V_{L}$ to $V_{H}$, the power $P_{PEH}$ must be greater than the quiescent power $P_{q}$ consumed by the voltage monitoring circuit ($P_{PEH}>P_{q}$). During the harvesting phase, the only current consumption is through the voltage detection unit of the STM32L0 microcontroller, which operates in sleep mode with a quiescent current consumption $I_{q}$ of ≈ 1 μA. The voltage $V_{stor}$ ranges between $V_{H}$ and $V_{L}$, for an average voltage $V_{stor\_avg}$ given by Equation (3).

(3) $$V_{stor\_avg}=\frac{V_{H}+V_{L}}{2}=2.5\;V.$$

The quiescent power consumption $P_{q}$ of the EAWSN during the harvesting phase is given by (4):

(4) $$P_{q}=V_{stor\_avg}\;\cdot \;I_{q}=2.5\;\mu W.$$

Therefore, the minimum power $P_{PEH\_min}$ that the PEH must generate at the minimum acceleration $a_{cc\_min}$ is given by (5):

(5) $$P_{PEH\_min}>\frac{P_{q}}{\eta_{rectfier}}$$

$\eta_{rectfier}$ is the power conversion efficiency of the full-bridge voltage rectifier, whose theoretical maximum value is equal to 8/$\pi^{2}$. Equation (5) shows the specification for the minimum power that PEH must generate when stressed by vibrations with minimum acceleration $a_{cc\_min}$, which is given by Equation (6).

(6) $$P_{PEH\_min}>\frac{P_{q}\;\cdot \;\pi^{2}}{8}\approx 3\;\mu W.$$

Another electrical parameter to specify for the PEH design is the PEH open-circuit voltage $V_{oc}$. The value of $V_{oc}$ is greater than the minimum supply voltage of the voltage detector circuit ($V_{dd\_min}$ = 1.8 V) and ensures the maximum power transfer between the PEH and the rest of the EAWVS system at the minimum acceleration $a_{cc\_min}$. Equation (7) defines the condition of $V_{oc}$ to achieve the maximum power transfer.

(7) $$\frac{V_{oc}-2\;\cdot \;V_{f}}{2}=V_{stor\_avg},$$

where $V_{f}$ is the forward conduction voltage of the voltage rectifier diode. From Equation (3) and Equation (7), and assuming a forward voltage drop $V_{f}$ of the diode of 0.6 V, Equation (8) provides the specification value for the PEH open-circuit voltage.

(8) $$V_{oc}=2\;\cdot \;V_{f}+V_{stor\_avg}=6.2\;V.$$

**Figure 3:**
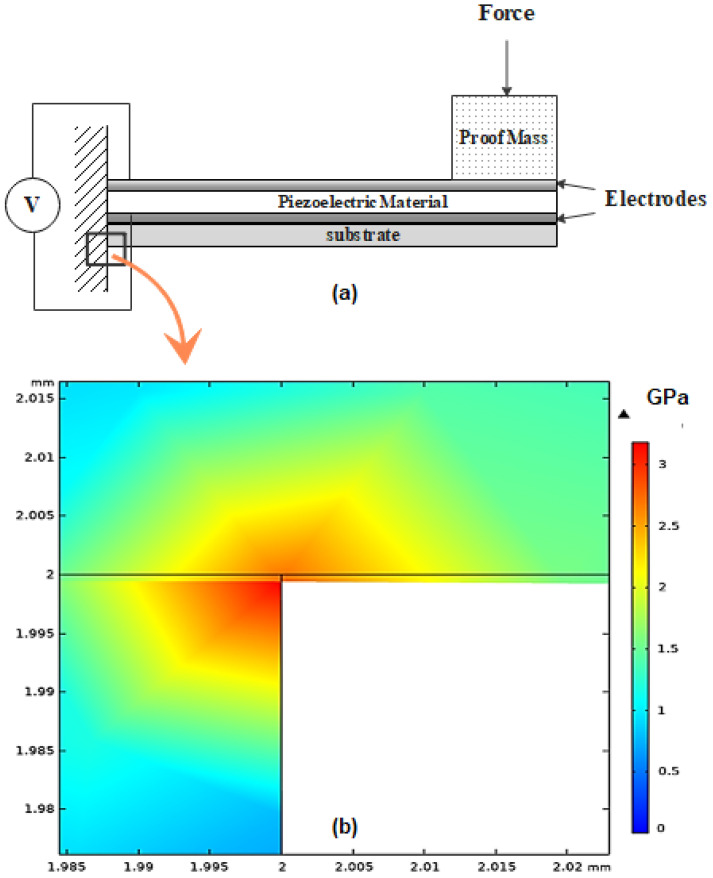
PEH cross sectional schematic and FEM analysis: (**a**) Cross sectional schematic of the PEH. (**b**) Zoom view of maximum mechanical stress distribution area obtained from FEM in the cantilever beam at $a_{cc}$ = 2 g.

**Figure 4:**
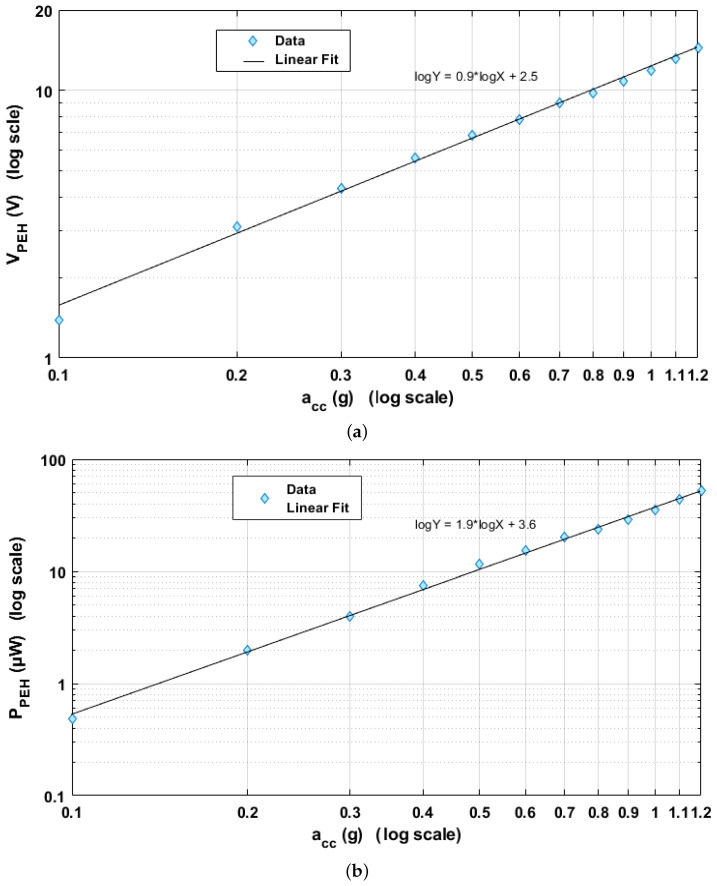
PEH experimental characterization as a function of acceleration $a_{cc}$: (**a**) Open-circuit voltage $V_{oc}$ in volts vs. acceleration $a_{cc}$ in g in log scale; (**b**) PEH generated power $P_{PEH}$ in μW vs. acceleration $a_{cc}$ in g in log scale.

The maximum detectable acceleration $a_{cc\_max}$ of the EAWVS relates to the highest stress level that the PEH substrate material can withstand (3–6 GPa for Si) [56]. The PEH form factor is another key aspect of the design phase as the typical specification is to implement a PEH that is comparable with the electronic circuit section in size [57]. Z-cut LN has been subjected to little investigation with respect to energy harvesting due to its low strain piezoelectric coefficient (d${}_{31}$ = −1 pm/V). Instead, the LN (YXl)/128${}^{\circ }$ single-crystal orientation presents much lower dielectric constant than PZT family ($\epsilon /\epsilon_{0}=50.5$) and higher piezoelectric coefficient compared with other Pb-free materials (d${}_{23}$ = 27 pm/V) [36]. The LN on silicon PEH is manufactured using Au-Au bonding technology developed by FEMTO-ST [37], in the form of a 1 cm${}^{2}$ unimorph clamped cantilever beam PEH. The active piezoelectric layer is a 30 μm thick LN (YXl)/128${}^{\circ }$ single crystal, which is fabricated to attain roughly 1.4 nF clamped capacitance for optimal electronic interfacing. The substrate is produced with a silicon wafer (500 μm thick), as illustrated in Figure 3a. The maximum limit of the input acceleration was determined to be ≈ 2 g from the Finite Element Method (FEM) simulations in COMSOL Multiphysics V5.5 as shown in Figure 3b. In this research, device testing was limited to 1.2 g, or 60% of the theoretical maximum acceleration, to ensure a safety margin for not exceeding the material stress reliability limits. The proof mass m attached to the cantilever beam allows tuning of the PEH resonance frequency $f_{0}$, which equates to 187 Hz in this design. At the resonance frequency $f_{0}$ and the minimum acceleration $a_{cc\_min}$ of 0.5 g, the PEH provides an open-circuit voltage $V_{oc}$ of 6 V and can supply a minimum power $P_{PEH\_min}$ of 12 μW, enough to compensate the quiescent power consumption $P_{q}$ and successfully charge the storage capacitor $C_{storage}$. The $V_{oc}$ was measured in the open-circuit condition with an oscilloscope and the RMS power $P_{PEH}$ of the harvester was obtained at the optimal load of 550 kΩ. Figure 4 shows the experimental measurements of the variation of $V_{oc}$ and $P_{PEH}$ as the acceleration $a_{cc}$ varies.

## 5. Vibrational Energy to Time Convertion

The PEH illustrated in Figure 3a, when subjected to the applied force F that induces the acceleration $a_{cc}$, generates the voltage $V_{PEH}$ across its electrodes in the d31 mode given by Equation (9) [58].

(9) $$V_{PEH}=\frac{d_{23}\;\cdot \;F\;\cdot \;l}{A\;\cdot \;\epsilon \;}=\left(\frac{d_{23}\;\cdot \;l\;\cdot \;m}{A\;\cdot \;\epsilon }\right)\;\cdot \;a_{cc}$$

The power $P_{PEH}$ generated on a load $Z_{load}$ when the PEH is excited by a harmonic oscillation is given by Equation (10) [59]

(10) $$P_{PEH}=\frac{V^{2}}{2\;\cdot \;Z_{load}}=\frac{1}{2\;\cdot \;Z_{load}}\;\cdot \;{\left(\frac{d_{23}\;\cdot \;l\;\cdot \;m}{A\;\cdot \;\epsilon }\right)}^{2}\;\cdot \;a_{cc}^{2}$$

- $d_{23}$ is the piezoelectric coefficient of lithium niobate (27 pm/V);
- *l* is the length of piezoelectric transducer (2 cm);
- *m* is the effective mass of the proof mass-cantilever system (2.3 grams);
- $a_{cc}$ is the amplitude of input acceleration;
- *A* is the area of the electrode (1cm${}^{2})$;
- $\epsilon /\epsilon_{0}$ is the relative dielectric permittivity of LN (YXl)/128${}^{\circ }$ (50.5).

During the harvesting phase, the load $Z_{load}$ is constant; hence, $P_{PEH}$ varies proportionally with the square of the acceleration $a_{cc}$, as given by Equation (11)

(11) $$P_{PEH}=K_{acc}\;\cdot \;a_{cc}^{2},$$

where, $K_{acc}$ is a constant of proportionality given by Equation (12)

(12) $$K_{acc}=\frac{1}{2\;\cdot \;Z_{load}}\;\cdot \;{\left(\frac{m\;\cdot \;d_{23}\;\cdot \;l}{A\;\cdot \;\epsilon }\right)}^{2}$$

The power harvested after each beacon in the $C_{storage}$ capacitor is given by Equation (13)

(13) $$P_{harvested}=\eta_{p}\;\cdot \;P_{PEH},$$

where $\eta_{p}$ is the power conversion efficiency of the EAWVS system when converting the power $P_{PEH}$ into the power harvested $P_{harvested}$ in the storage capacitor $C_{storage}$. By combining Equations (11) and (13), the power $P_{harvested}$ can be related to the acceleration $a_{cc}$ as in (14)

(14) $$P_{harvested}=\eta_{p}\;\cdot \;K_{acc}\;\cdot \;a_{cc}^{2}$$

Equation (15) relates $P_{harvested}$, $E_{harvested}$ and the time $t_{rise}$ that it takes to charge the $C_{storage}$ capacitor from the voltage $V_{L}$ to voltage $V_{H}$ during the energy-harvesting phase.

(15) $$P_{harvested}=\frac{E_{harvested}}{t_{rise}}$$

The EAWVS communicates with the BS via the BLE wireless protocol by periodically transmitting beacons of duration $t_{adv}$, which is the advertising time that elapses between successive beacons. The time $t_{adv}$ is remotely measured by the BS while receiving the beacons and is used to indirectly measure the acceleration $a_{cc}$ induced on the PEH. The time $t_{rise}$ is related to the advertising time $t_{adv}$, as in Equation (16).

(16) $$t_{rise}=t_{adv}-t_{fall}$$

The fall time $t_{fall}$ is related to the parameters of the system, $V_{H}$, $V_{L}$, $C_{storage}$ and $I_{ble}$, as in Equation (17).

(17) $$t_{fall}=\frac{C_{storage}\;\cdot \;\left(V_{H}-V_{L}\right)}{I_{ble}}$$

The parameter $I_{ble}$ represents the average current consumption of the BLE radio during the transmission phase and has a constant value of ≈9 mA for the BLE radio configured in non-connectable advertising mode to transmit an output power of 8 dBm and a data length of 20 bytes. Therefore, the time $t_{fall}$ is also constant, and equates to ≈45 ms in this implementation. By combining Equations (2), (13)–(16) and Equation (18), one can see how the acceleration $a_{cc}$ can be indirectly derived from the measurement of the time $t_{adv}$.

(18) $$a_{cc}^{2}=\frac{C_{storage}\;\cdot \;\left(V_{H}^{2}-V_{L}^{2}\right)}{K_{acc}\;\cdot \;\eta_{p}\;\cdot \;\left(t_{adv}-t_{fall}\right)}$$

**Figure 5:**
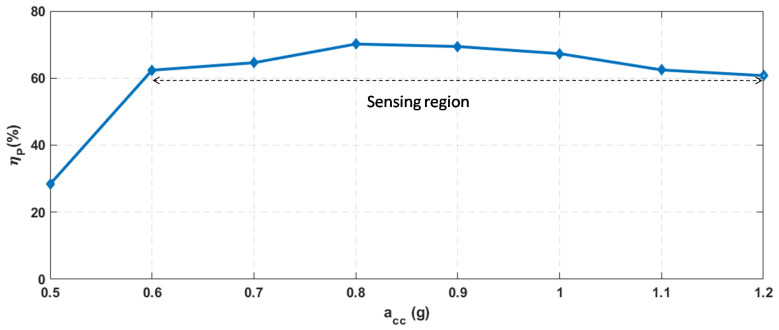
EAWVS power conversion efficiency $\eta_{p}$ vs. acceleration $a_{cc}$ (experimental results).

The parameter $\eta_{p}$ is specific to the EAWVS system and is derived experimentally from its post-production characterization. Figure 5 shows the experimental characterization of the parameter $\eta_{p}$ while varying the acceleration $a_{cc}$. At 0.5 g, the PEH provides just enough power to sustain the system, and the $\eta_{p}$ at this acceleration is low. The PEH provides more power with increasing $a_{cc}$, such that $\eta_{p}$ becomes stable for accelerations above 0.6 g. The high initial variation of $\eta_{p}$ at lower acceleration levels between 0.5 g, and 0.6 g affects the linearity of the EAWVS in its sensor function. Therefore, even though the EAWVS remains self-powered from an input acceleration of 0.5 g, the system efficiency is so low and variable that the use of the EAWSN as a sensor is more or less compromised. Therefore, the LoD of the EAWVS is set at the higher level of 0.6 g, where the $\eta_{p}$ is higher and remains almost constant at the average value of 65% with a max variation <±8% in the acceleration range [0.6 g–1.2 g].

Since $\eta_{p}$ remains nearly constant within the acceleration range [0.6 g–1.2 g], Equation (18) can be rewritten as:

(19) $$a_{cc}=\frac{S_{v}}{\sqrt{\left(t_{adv}-t_{fall}\right)}},$$

where $S_{v}$, given by Equation (20), is the sensitivity of the EAWVS.

(20) $$S_{v}=\sqrt{\frac{C_{storage}\;\cdot \;\left(V_{H}^{2}-V_{L}^{2}\right)}{K_{acc}\;\cdot \;\eta_{p}}}$$

Furthermore, the high imbalance between the current produced by the PEH during the harvesting phase (μA) and that consumed by the EAWVS during the transmission phase (mA) results in a large difference between $t_{adv}$ (s) and $t_{fall}$ (ms). Hence, it is reasonable to assume that in a real application scenario, $t_{adv}\gg t_{fall}$, implying that the following approximation $t_{adv}-t_{fall}\approx t_{adv}$ is possible for negligible errors, so Equation (19) can be simplified as Equation (21).

(21) $$a_{cc}\approx \frac{S_{v}}{\sqrt{t_{adv}}}$$

Equation (21) is easily implemented in the embedded firmware of the BS, so the acceleration $a_{cc\_BS}$ can be remotely and indirectly measured at the BS that can measure the time $t_{adv}$ from the received beacons. It is worth noting that, since the EAWVS exploits measurements in the time domain, the data reading interface are inherently digital. This feature is very convenient as it involves less complicated electronics for data reading than analog architectures and, more importantly, a lower energy consumption, which is a fundamental criterion of energy-autonomous and battery-free systems [60]. Since the BS can measure the time $t_{adv}$, the only remaining parameter required to calculate the acceleration $a_{cc\_BS}$ via Equation (21) is $S_{v}$, which is derived experimentally through the characterization of the EAWVS in Section 6.

## 6. Experimental Setup and Measurements

**Figure 6:**
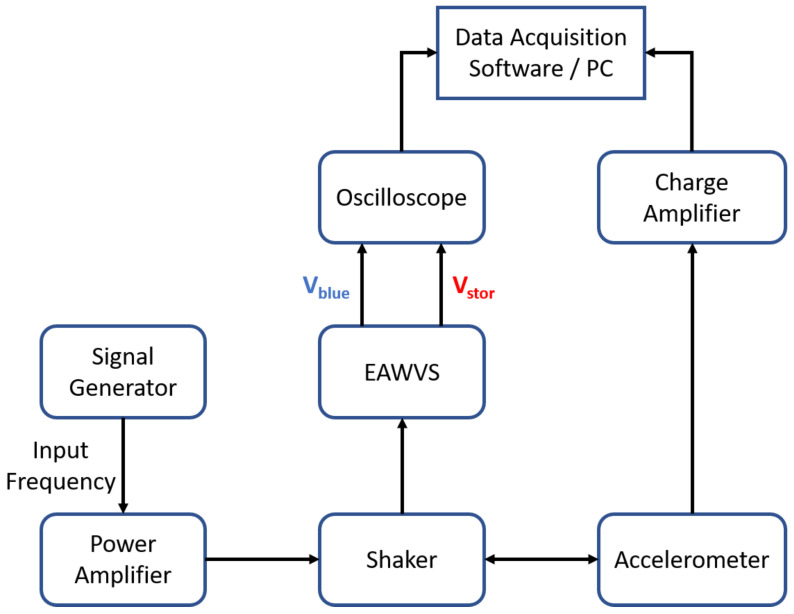
Block diagram of the experimental setup.

Figure 6 shows the block diagram of the experimental setup, which consists of:

- A signal generator: this equipment (Agilent 3500B) is used to control the input frequency and the vibration amplitude of the shaker;
- Power amplifier: a 100 W power amplifier (PA 100E Data Physics) that amplifies the signal provided by the signal generator;
- Shaker: the electrodynamic shaker (SignalForce from Data Physics) provides the input vibration for the PEH;
- Accelerometer and charge amplifier: these devices are implemented with the PCB Piezotronics 355B04 accelerometer. They are connected to the shaker to measure the input acceleration in real time.

**Figure 7:**
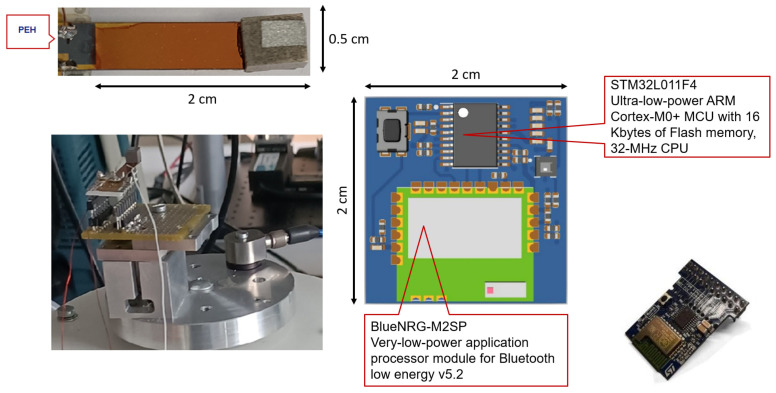
Experimental setup, EAWVS PCB and PEH.

**Figure 8:**
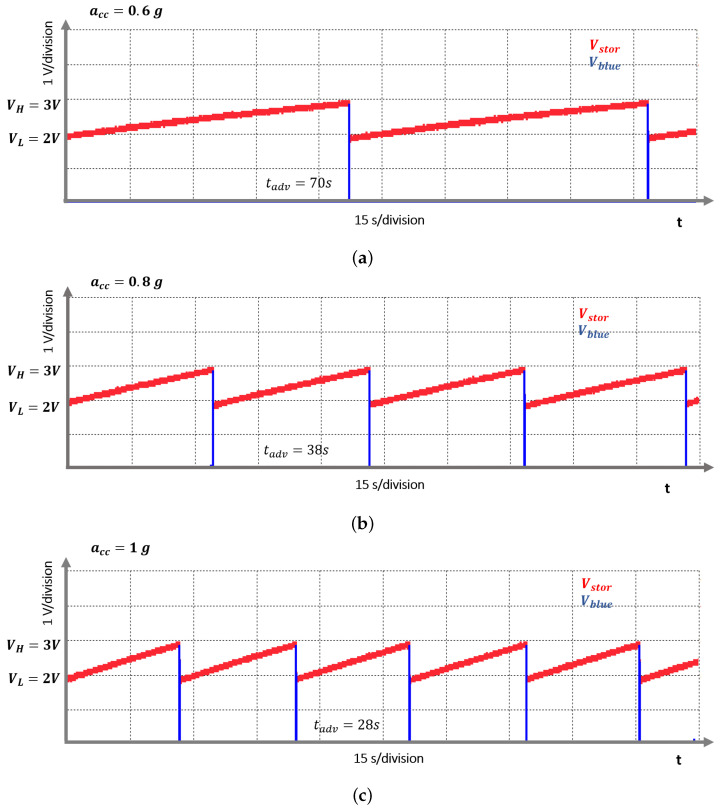
$V_{stor}$ and $V_{blue}$ vs. time at three different accelerations: (**a**) $a_{cc}$ = 0.6 g, (**b**) $a_{cc}$ = 0.8 g and (**c**) $a_{cc}$ = 1 g.

Figure 7 shows the experimental setup, the top view and layout of the PCB that implements the EAWVS, and the top view of the PEH. The EAWVS characterization in terms of LoD and sensitivity in response to the vibrational acceleration was carried out at room temperature in ambient air. The EAWVS was clamped on the shaker and was excited with amplitude of acceleration $a_{cc\_in}$ in the range [0.5 g–1.2 g]. Figure 8 shows several measurements of the output voltage $V_{blue}$ and the corresponding time $t_{adv}$, probed with an oscilloscope for the various acceleration levels. Figure 8 shows how the time $t_{adv}$ decreases with the increase in acceleration $a_{cc}$, in agreement with Equations (18), (19) and (21).

**Figure 9:**
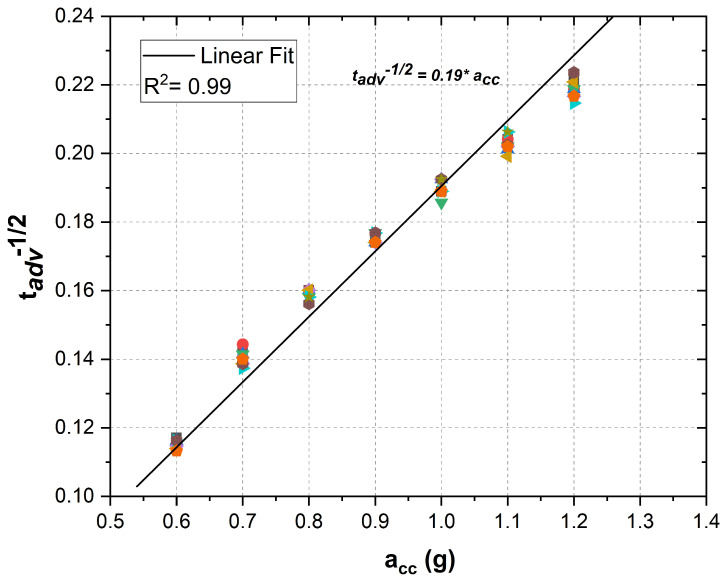
Linear fit of the experimental measurement data for various accelerations from 0.6 g to 1.2 g.

The EAWVS sensitivity $S_{v}$ is the inverse of the fitting line slope reported in Figure 9. Hence, from the EAWVS characterization, $S_{v}\;=\;5.26$ g · s${}^{1/2}$; therefore, the acceleration at the BS can be indirectly measured by knowing $S_{v}$ and measuring time $t_{adv}$ using Equation (22).

(22) $$a_{cc\_BS}=\frac{5.26}{\sqrt{t_{adv}}}$$

The intrinsic error with the proposed measurement technique is indicated with $Error_{acc}$ and calculated through Equation (23), representing the acceleration measurement error between the acceleration $a_{cc\_BS}$, indirectly measured at the BS and the forced acceleration $a_{cc\_in}$ at the EAWVS.

(23) $$Error_{acc}=100\;\cdot \;\left|\frac{a_{cc\_in}-a_{cc\_BS}}{a_{cc\_in}}\right|$$

**Figure 10:**
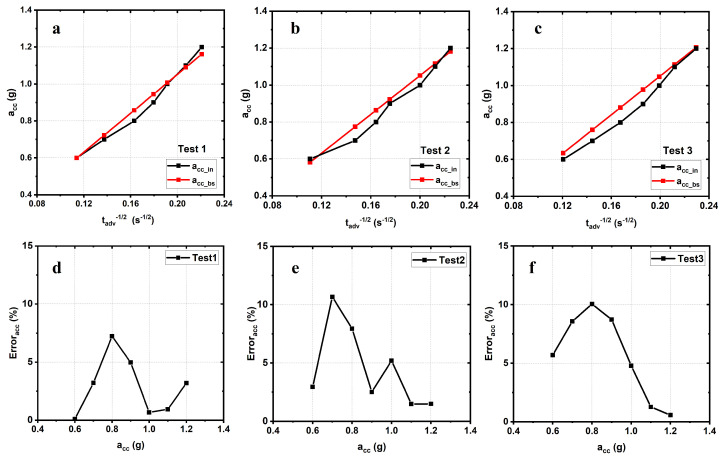
(**a**–**c**) $a_{cc\_in}$ and $a_{cc\_BS}$ vs. $t_{adv}^{-1/2}$ for three tests to study reproducibility. (**d**–**f**) Measurement of corresponding $Error_{acc}$ vs. the input acceleration $a_{cc\_in}$ for three different tests.

Figure 10a–c show the variation of $a_{cc\_in}$ and $a_{cc\_BS}$ vs. $t_{adv}$. Figure 10d–f shows the variation of the error $Error_{acc}$ as the input acceleration $a_{cc\_in}$ varies over the range [0.6 g–1.2 g]. It is worth highlighting that the discrepancy reported between the measured data reported in Figure 10d–f and the theoretical from Equation (22) is due to the variation of the system’s energy-harvesting efficiency which is not constant along with the given acceleration range as reported by the results shown in Figure 5.

The test measurements confirm that a remote and wireless measurement of the acceleration forced onto the EAWVS is possible at the BS. The measurement results are repeatable with an error consistently below 11%.

By observing Figure 10, it is worth considering that the error has a wide variation in the considered acceleration range that is mainly due to the efficiency variation on the system power conversion efficiency $\eta_{p}$. Therefore, to implement a better approximation, one of the strategies employs a piecewise approximation [54]. The whole acceleration range [0.6 g–1.2 g] is divided in two sub-ranges, [0.6 g–0.9 g] and [0.9 g–1.2 g]. In the operating range [0.6 g–0.9 g], it is possible to calculate the acceleration at the BS through (24), which represents the linear regression, with an error lower than ±7.3%, while in the sub-range [0.9 g–1.2 g], it is possible to calculate the acceleration at the BS through (25), with an error lower than ±6.8%.

(24) $$a_{cc\_BS}=\frac{5.1}{\sqrt{t_{adv}}}\;\;\;\;\;\;\;\;\;\;\;\;with\;a_{cc\_BS}\in \left[0.6\;g–0.9\;g\right]$$

(25) $$a_{cc\_BS}=\frac{5.34}{\sqrt{t_{adv}}}\;\;\;\;\;\;\;\;\;\;\;\;with\;a_{cc\_BS}\in \left[0.9\;g–1.2\;g\right]$$

Equations (24) and (25) can be implemented in the firmware of the BS that continuously monitors the advertising time and calculates the acceleration $a_{cc\_BS}$. Table 1 reports a summary of the equations and related errors.

**Figure 11:**
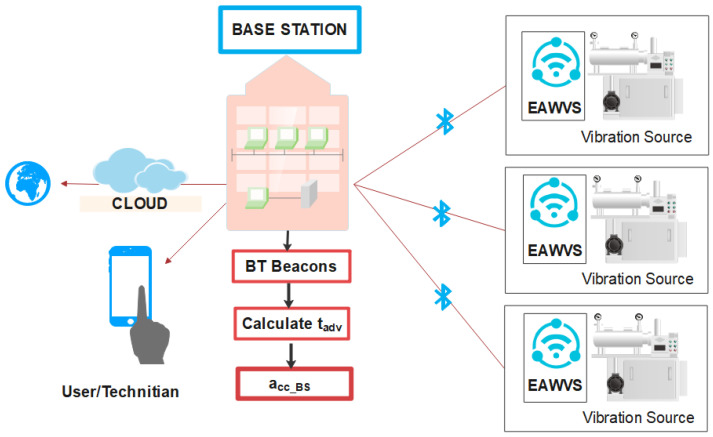
Application scenario with BS and several EAWVSs.

The BS can therefore receive beacons from one or more EAWSNs placed on the vibrating components of machines under test and implement critical predictive maintenance or fault detection functionality by measuring the advertising time $t_{adv}$ of the received beacons as shown in Figure 11. For the long-term predictive diagnosis of machines, the EAWVS can be embedded on specific mechanical parts that require regular maintenance, such as bearings, gears, conveyors, turbines, shafts, valves, etc.

Experiments have also been carried out with a remote BS. The BS was implemented with the BLUENRG-2 IC set to scanning mode to receive the data transmitted by the battery-free EAWVS, configured in advertising non-connectable mode to transmit output power of 8 dBm. The EAWVS and the BS were placed at an average distance of 5 m. During the measurements, several other devices using Bluetooth and Wi-Fi were active, including a 2.4 GHz Wi-Fi access point and various personal computers and smart devices. The EAWSN and BS have also been tested in a free space environment reaching a reliable communication distance of 100 m.

## 7. Conclusions

This paper studied a novel self-powered and battery-free device for vibration sensing based on the conversion of vibration energy to time data. The underlying principles and physical equations form the basis of a measurement technique using temporal data to determine the amplitude of vibrational acceleration experienced by an object. The proposed approach generates remote measurement readouts at a designated base station (BS) from the wireless BLE transmission of sensor data expressed in easy-to-implement time-domain readouts. The design of a specially designed lead-free PEH able to perform the double function of energy harvester and vibrational sensor is integral to the proposal, and the experimental results demonstrate reliable sensing in the acceleration range [0.6 g–1.2 g]. Experimental tests have shown that the EAWVS and BS system can deliver repeatable and accurate sensing functionality with less than 11% maximum error. It has been evidenced how the simplicity of the EAWVS architecture translates into several advantages, such as eco-friendliness, size, cost and, above all, the capacity to function as a maintenance-free install-and-forget device in practically any application where vibration energy is available. Finally, all experimental measurements were performed using a 2 cm × 2 cm PCB with a thickness of 0.45 cm, implemented (except for the PEH) with standard components to demonstrate the feasibility of a proof of concept, the operational performance of which can only improve with advances in relevant materials and electronic components. Our aim is to therefore build on the encouraging results reported herein with further research into more advanced materials and architectures that can ensure greater versatility and applicability across an even wider range of use case scenarios.

## Figures and Tables

**Table 1:** Experimental results with maximum error in measurement for two different acceleration rages.

$a_{\mathrm{cc}}$ Range (g)	Fitting Equation	Max ${\mathrm{Error}}_{\mathrm{acc}}$ (%)		
		Test 1	Test 2	Test 3
[0.6–0.9]	$a_{cc\_B\_S}=\frac{5.1}{\sqrt{t_{adv}}}$	3.9	7.3	6.7
[0.9–1.2]	$a_{cc\_B\_S}=\frac{5.34}{\sqrt{t_{adv}}}$	6.5	6.8	5.4
